# 

**Published:** 1888-11-24

**Authors:** 


**Notice to Advertisers and Subscribers.**

All Advertisements, Orders for Copies of The Hospital, and Business Communications, should be addressed to The Publisher, *not to the Editor*, The Hospital, 38, Old Jewry, E.C.

A Scale of Charges will be found in the Advertisement Sheets, page iii.

**To Residents, Secretaries, and Matrons.**

The Editor will be glad to have the Regulations and each Report as issued by Asylums, Hospitals, Convalescent and Nursing Institutions, as well as those of other Charities, and an early copy in duplicate of all Appeals and Festival Notices, etc., addressed to *The Editor, The Lodge, Porchester Square, London, W.* Any superintendent, secretary, matron, or other chief official who regularly sends such information may be registered, on application to the Publisher, to receive free of cost a cover for binding The Hospital in half-yearly volumes.

### Scraps and Gleanings.

There were 34 deaths from diphtheria in London last week.

Mr. Victor Horsley is one of Pasteur's most ardent disciples in this country.

Hospital Sunday at Salisbury resulted in £203 against £201 collected last year.

The Right Hon. Lord Gerard is appointed president of Providence Free Hospital.

Cork Street Fever Hospital, Dublin, issues an appeal for old clothes for patients.

Northampton General Hospital has published a copy of the proposed revised rules.

Mr. Arthur G. Morrison has written an account of the London Hospital for *The People*.

The Emperor William is suffering from neuralgic headaches and purulent catarrh of the left ear.

Mr. Thomas Hope has intimated his intention of building and endowing a hospital at Langholm.

Mr. Alfred Cooper has resigned his post of surgeon to the out-patients of the London Lock Hospital.

Dr. J. MacWeeney has been appointed Pathologist to the Mater Misericordæ Hospital, Dublin.

The Great Northern Central Hospital had to refuse admittance to 119 patients last week, for want of room.

Five guineas was found in the Hospital box at St. John's Sunday Schools, Devonport, when it was last opened.

Mr. Walters has collected the necessary £16 for furnishing one bed at the Wallingford Cottage Hospital.

Last Saturday the usual Popular Evening Entertainment for the People at Richmond was in aid of the hospital.

Mr. Labouchere's annual show of toys for distribution in hospitals and workhouses will open on December 20th.

It is said that St. Helen's Cottage Hospital is the only hospital in Great Britain with a balance on the right side.

Lady Armstrong has offered to plant with shrubs, the ground adjoining the Fleming Memorial Hospital, Newcastle.

The usual weekly entertainment was given at Brompton Hospital on Tuesday last by Miss May Woolgar-Melton.

Mr. Frederick Treves is delivering a course of lectures to nurses on Wednesday evenings at the London Hospital.

Professor Bamberger, one of the most distinguished representatives of the Vienna School of Medicine, died on Monday last.

The Society for the Study of Inebriates in its November report, speaks encouragingly of success in securing a cure in the future.

One hundred and ninety-six patients attended the British Hospital for Diseases of the Skin during the week ending November 10th.

St. Barnabas Cottage Hospital, Saltash is nearly completed. It is a beautiful building erected by Mrs. Ley, in memory of her husband.

Mr J. E. Greaves, Lord-Lieutenant of the County of Carnarvon, has been elected president of the Carnarvonshire and Anglesey Infirmary.

The annual meeting of the Committee of the Royal Hospital for Incurables will take place on November 30th, Mr. J. D. Allcroft in the chair.

Many European women have left the Lying-in Hospital, Calcutta, because of a new rule allowing native students to attend them in their confinements.

The architect and builder of the new hospital at Berlin, part of which fell and killed eight men in the spring, have both been condemned to six months imprisonment.

A fire, which broke out in the ancient Castle of Libenwerda, near Halle, burned to death the medical practitioner of the district, who occupied a wing of the building.

Dr. Sykes is trying to prevent further buildings being erected on the site of Poplar Place, near the Foundling Hospital. He says the district is already over-crowded.

It is suggested to use the house near Stockwell Fever Hospital which is at present the subject of much discussion, as a home for the nurses who are now lodged "in the roof."

There are still thirty patients in the Infectious Hospital Woodborough Road, Nottingham, and the scheme for erecting another hospital without the town makes no advance.

Many of the medical men of Hull object to the foundation of an orthopædic hospital in that town. They believe thorough treatment can be secured in the general hospital.

Dr. Freyer recently received a fee of 112 000 rupees for medical attendance on the Nawab of Rampore. The Government has ordered part of the fee to be returned.

At the last examinations of the Sanitary Institute, six out of fifteen candidates took the Surveyor's Certificate, and 31 out of 59 candidates took the Inspector of Nuisances Certificate.

Lady Lansdowne will take over the presidentship of Lady Dufferin's Fund on her arrival in India, at which time it is expected there will be a balance of 30,000 rupees in hand besides the capital.

A grand matinée will be given at the Avenue Theatre on December 13th, in aid of the Samaritan Fund of the Middlesex Hospital. Count Arnheim will conduct, and numerous well-known artistes have offered their services.

Mr. E. H. Douty, M.A., of King's College, has been appointed Senior Demonstrator in Anatomy at Cambridge University. Mr. W. S. Melsome, B.A., Fellow of Queen's College, and Mr. R. W. Michell, B.A., of Gonville and Caius College, have been appointed Junior Demonstrators of Anatomy.

Yellow fever is officially declared to have broken out in Santo Cruz, the chief town in the island of Palma. The disease was imported by a vessel from Cuba. Communication has been completely cut off between Palma and the other islands. The public health in Grand Canary continues excellent.

### Amusements and Relaxation.

### NEW PUZZLE PRIZE SCHEME.

COMMENCED OCTOBER 6TH, ENDS DECEMBER 29TH, 1888.

N.B.—All contributions must reach the office, 38, Old Jewry, E.C., not later than the *First Post* on *Thursday* in each week.

### PUZZLES FOR SOLUTION.

**Sixth Week.**

No. 16. Topical Alphabet.

A. is the —— who turns a cart wheel.
The Board School could not excite the same zeal.
B. is for —— with his Parachute.
Is it a gain, or will science refute?
C. is the —— that was issued last year.
'Twas not at all good, the Queen's so severe.
D. is for —— "Thou hast done well."
The "Women of India" have much to tell.
E. is for ——, that beautiful land
Where misery's wrought by the Parnellite band.
F. is for —— with his gunpowder scare,
A warning to us, of Fenians beware!
G. is the —— we should teach by our lives,
For preaching too much is not very wise.
H. is our ——, then think of the poor,
And drop in your coin at the hospital door.
I. is for —— our distant possession,
Russia is long in learning that lesson.
J. is for —— do the Board of Works know
The science that ends with the words "Hey, Presto!"
K. is the —— which opens the door,
It's often of gold, to make it more sure.
L. is the —— the Home Rulers would break,
Pretending it is for dear "Ould Ireland's sake."
M for the —— that met in the square,
A pity! the lions didn't give them a scare.
N. for the —— who *will* make a speech,
Although of the law, he knows it's a breach.
O. for the —— of which there are several,
May be for soups, or a m jo general.
P. for —— the invisible man,
Whenever he's wanted, find him who can?
Q. is the —— now asked by the man,
'Twill soon be the woman alone who can.
R. is the —— which we hear of so much,
Where will the line be drawn they mustn't touch?
S. is for —— the sun that is clear,
Skipped over summer, because 'twas leap year.
T. is for "all —— " meaning eternity.
Use it well, make the future a certainty.
U. is for —— true patriotism,
Whether in Liberal, or Conservatism.
V. is the —— at which we cry shame.
Do we do our best, or are we to blame?
W. is for ——, grim want is near,
No prospect of work! as it was last year.
X. is for —— the athletic's delight,
Where he likes to show off in a "glove" fight.
Y. is the —— which comes to an end,
With our greetings for Christmas to many a friend.
Z. is the —— applied to this jingle,
In which topics grave and gay variously mingle. Gretta.

No. 17. Charade.

My *first* is a small but useful word,
A preposition often heard.
My *second's* a tree, both stately and tall,
From which in autumn, the brown cones fall.
My *third* is the name of Scotland's fair Queen,
Whose mournful fate often told hath been.
My *whole* is a place where those who are ill,
Are tended by nurses and doctors with skill.
John Gilpin.

**Marks.**

| Names. | No. of Puzzles sent in Nov. 15. | No. of Marks Nov. 15 | Total of Marks. | Names. | No. of Puzzles sent in Nov. 15. | No. of Marks Nov. 15. | Total of Marks. |
|---|---|---|---|---|---|---|---|
| Patience .... | 3 | 4 | 32 | Hercules .. | 3 | 0 | 11 |
| Lady Clara.. | 3 | 6 | 37 | Gretta ...... | 3 | 6 | 22 |
| Tinie ...... | 3 | 7 | 30 | Dandy Dick | — | — | 5 |
| Bijou ...... | 2 | 4 | 15 | Boadicea .. | — | — | 7 |
| G. D. ...... | 3 | 4 | 15 | Nipper .... | 2 | 5 | 23 |
| Thanet .... | 2 | 4 | 22 | Morico .... | 3 | 7 | 18 |
| Daisy ..... | 1 | 0 | 11 | A. M. E..... | — | — | — |
| Salford .... | — | — | 6 | John Gilpin | — | — | — |

**Answers to Fourth Week Puzzles.**

No. 10 Double Acrostic.

N in E
O i L
V ers E
E lectri C
M aggo T
B arzilla I
E ch O
R easo N

No. 11. Decapitation. Emigration, Migration, Ration.

No. 12. Charade. Par-(k)nell = Parnell.

**Marks for Solution of Puzzles.**

Total Marks.—Ruffles (2), 6; Daisy (1), 5; Lady Clara (2), 8; Reldas, (1), 6; Mater (1), 4; Condorcet (1), 5; Tinie (1), 6; Gentian (2), 7; Marguerite, 5; Patience (1), 3; Gretta 3; Amicus (2), 4; Scrooge (2), 7; Smut, 2; Thanet (3), 10; Jenny Wren, 1.

*The figures in parentheses are Marks for the week ending Nov. 15.*

**Notices to Correspondents.**

*Hercules and Daisy.*—Marks are not given, unless the rule heading this column last week "Prizes will be awarded, etc.," be fully carried out.

(*For Conditions, see last week's* The Hospital.)

